# Brooke-Spiegler Syndrome with Multiple Scalp Cylindromas and Bilateral Parotid Gland Adenomas

**DOI:** 10.1155/2012/249583

**Published:** 2012-02-12

**Authors:** Peter Kalina, Rokea el-Azhary

**Affiliations:** ^1^Department of Radiology, Mayo Clinic, Rochester, MN 55905, USA; ^2^Department of Dermatology, Mayo Clinic, Rochester, MN 55905, USA

## Abstract

A 62-year-old female presented with numerous soft tissue lesions of her scalp and bilateral preauricular region. Several of these have been biopsied or removed with a diagnosis of cylindromas. Cylindromas are benign tumors with a differentiation towards apocrine sweat glands that increase in number and size throughout life. Multiple scalp cylindromas may coalesce and cover the entire scalp, resulting in the “turban tumor.” These are often associated with the autosomal dominant Brooke-Spiegler syndrome with coexistent facial trichoepitheliomas and spiradenomas. There is a very rare association between cylindromas and basal cell adenoma and adenocarcinoma of the parotid gland, with only 17 reported cases. Ours is the first CT demonstration of both the scalp and parotid gland findings in this uncommon situation.

## 1. Introduction

Cylindromas are benign tumors histologically similar to sweat glands. Multiple scalp cylindromas may coalesce to cover the entire scalp, resulting in the so-called “turban tumor.” Multiple scalp cylindromas are often associated with Brooke-Spiegler syndrome. There is a rare association between cylindromas and basal cell adenoma as well as adenocarcinoma of the parotid gland. This is the first case to demonstrate the CT appearance of both the scalp and parotid gland findings in this uncommon syndrome.

## 2. Case Report

**Figure 1:**
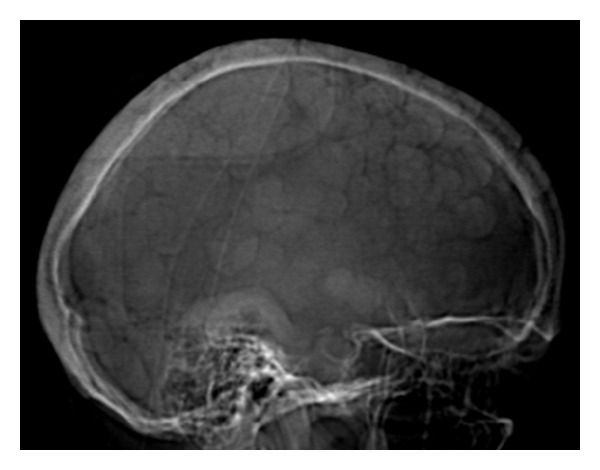
Skull radiograph, lateral view: multiple nodular densities overlie the calvarium.

**Figure 2:**
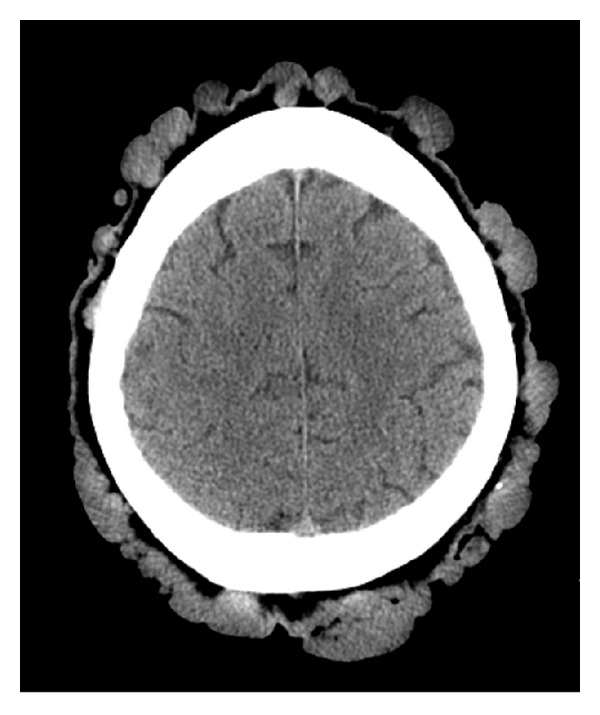
Noncontrast head CT: multiple nodular smooth surfaced well-demarcated soft tissue lesions arising from the scalp.

**Figure 3:**
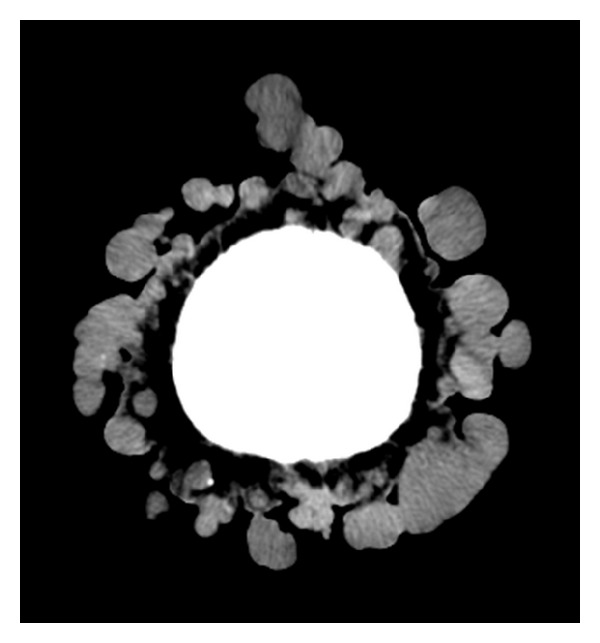
Noncontrast head CT: multiple nodular smooth surfaced well-demarcated soft tissue lesions arising from the scalp.

**Figure 4:**
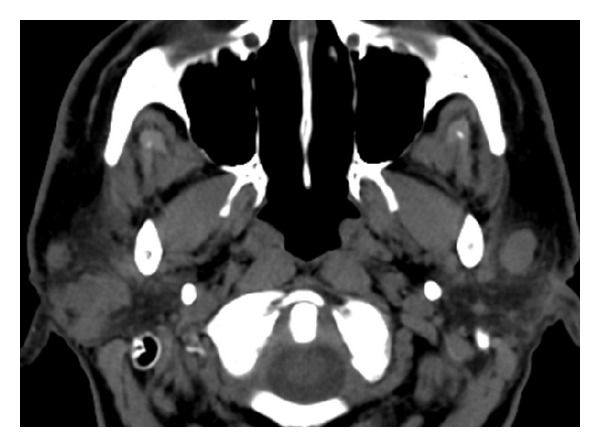
Head CT, noncontrast: multiple nodular masses of varying sizes in both parotid glands.

**Figure 5:**
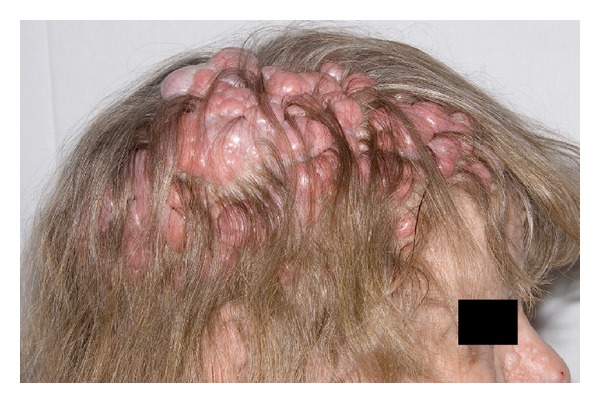
Clinical photographs of the multiple scalp lesions.

**Figure 6:**
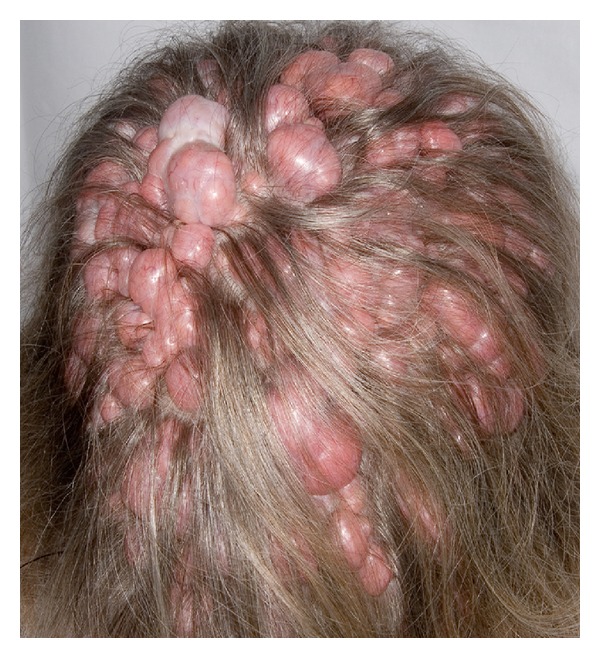
Clinical photographs of the multiple scalp lesions.

A 62-year-old female presented with a long history of numerous soft tissue lesions bulging from her scalp as well as her preauricular (parotid) region. These were noted to range in size from 2 mm to 2 cm diameter (Figures 1–6). Multiple similar appearing facial lesions were also noted. Several of these lesions have been biopsied or removed in the past. Unfortunately, many have continued to grow and many have recurred.

## 3. Discussion

Cylindromas are benign tumors that histologically demonstrate a differentiation towards apocrine sweat glands. They usually begin to appear in the second or third decades and tend to gradually increase in number and size throughout life [1, 2]. Multiple scalp cylindromas may coalesce and cover the entire scalp, resulting in the so-called “turban tumor.” These are typically associated with hair loss. The presence of multiple scalp cylindromas is often associated with the autosomal dominant Brooke-Spiegler syndrome, a condition in which there are coexistent facial trichoepitheliomas and spiradenomas [3]. This syndrome is caused by mutations in the tumor suppressor CYLD gene localized to chromosome 16q [1, 3]. Brooke-Spiegler syndrome tends to affect women more frequently than men. Although usually benign, rare transformation of cylindromas to malignant cylindrocarcinomas has been described. There is a very rare association between cylindromas and basal cell adenoma as well as adenocarcinoma of the parotid gland. However, only 17 such cases have been reported [4]. Ours is the 18th and represents the first case to demonstrate the CT appearance of both the scalp and parotid gland findings in this uncommon syndrome.
